# Serum galectin‐3 as a biomarker for screening, early diagnosis, prognosis and therapeutic effect evaluation of pancreatic cancer

**DOI:** 10.1111/jcmm.15775

**Published:** 2020-09-04

**Authors:** Nan Yi, Xuying Zhao, Jie Ji, Minxue Xu, Yujie Jiao, Tianyang Qian, Shengze Zhu, Feng Jiang, Jianhua Chen, Mingbing Xiao

**Affiliations:** ^1^ Department of Gastroenterology, Research Center of Clinical Medicine Affiliated Hospital of Nantong University Nantong China; ^2^ Department of Endocrinology, Research Center of Clinical Medicine Affiliated Hospital of Nantong University Nantong China; ^3^ Medical College Nantong University Nantong China; ^4^ Chinese Medicine 193 First Clinical Medical School Nanjing University of Chinese Medicine Nanjing China; ^5^ Department of Oncology Shanghai Tenth People's Hospital Tongji University Shanghai China; ^6^ Tongji University Cancer Center Shanghai China; ^7^ Department of Oncology Shanghai Dermatology Hospital Tongji University Shanghai China

**Keywords:** diagnosis, galectin‐3, pancreatic cancer, prognosis, screening

## Abstract

Galectin‐3 plays an important role in cell‐cell adhesion, macrophage activation, angiogenesis, metastasis and apoptosis and is overexpressed in pancreatic cancer. We explored the importance of galectin‐3 in the screening, early diagnosis, prognosis and therapeutic effect evaluation of pancreatic cancer. A time‐resolved fluorescence immunoassay was performed to detect serum galectin‐3 level. Serum samples were collected from healthy controls and patients with pancreatic cancer before and after different treatments, and the relationships between galectin‐3 level and clinical parameters were analysed. Among the healthy controls, one individual with an abnormally high concentration of galectin‐3 (9.85 μg/L) was diagnosed with pancreatic cancer. Compared to the pre‐operative level, galectin‐3 concentration significantly decreased in patients with radical excision 1 month after surgery (*P* < .05), but showed no obvious change in patients who underwent palliative resection. Additionally, among patients with radical excision, carcinoma recurrence rate was significantly higher in those with increased or unchanged galectin‐3 level. Retrospective analysis revealed the extraordinarily high value and high specificity of galectin‐3 for predicting 3‐year survival (*P* < .001). Thus, galectin‐3 may serve as a potential biomarker for the screening and early diagnosis of pancreatic cancer and as an independent prognostic indicator in patients with pancreatic cancer.

## 1 INTRODUCTION

Pancreatic cancer is a common malignant tumour of the digestive system. While the incidence of pancreatic cancer is increasing year by year, the associated mortality rate is predicted to rise and become the second highest among all malignant tumours by 2030.^1^ Given the high degree of malignancy, poor prognosis, low survival rate and lack of clinical features, the diagnosis and treatment of pancreatic cancer are extremely difficult. At present, surgical resection combined with post‐operative chemotherapy is the most effective regimen for pancreatic cancer treatment, but early diagnosis is still a serious concern. Tumour infiltration and distant metastasis may result in reduced surgical resection rate and poor response to chemotherapy.^2^, ^3^ Prognosis is greatly improved upon cancer diagnosis at an early operable stage. Until now, even recognized serum markers for pancreatic carcinoma, such as carcinoembryonic antigen (CEA) or carbohydrate antigen 19‐9 (CA19‐9), have not completely satisfied the criteria for early diagnosis. Therefore, it is imperative to develop more accurate and effective biomarkers for the early diagnosis, prognosis and therapeutic effect evaluation of pancreatic cancer.

Galectin‐3 is an important member of the β‐galactoside–binding protein family that is involved in cell‐cell adhesion, cell‐matrix interaction, macrophage activation, angiogenesis, metastasis and apoptosis.^4^, ^5^, ^6^ Many studies have shown the expression of galectin‐3 in various tumours, such as those of the gastrointestinal tract, cardiovascular, urinary and respiratory systems, breasts and thyroid gland.^7^, ^8^, ^9^, ^10^ Galectin‐3 overexpression is closely related to the proliferation, invasion and malignancy of tumour cells.^11^, ^12^, ^13^ In our previous proteomic study,^14^, ^15^ galectin‐3 is overexpressed in pancreatic carcinoma tissues. A strong galectin‐3 staining was predominantly observed in the cytoplasm and weak staining in the nucleus of tumour cells. Galectin‐3 highly expressed in pancreatic cancer tissue was also found to be secreted into the peripheral blood. Serum galectin‐3 level was higher in pancreatic cancer patients than in healthy individuals or patients with benign pancreatic lesions, periampullary tumours or acute pancreatitis. Galectin‐3 may serve as a diagnostic marker for pancreatic cancer. However, the relationship between serum galectin‐3 level and clinicopathological parameters remains elusive. Furthermore, we discussed the role of galectin‐3 in the assessment of therapeutic effect and prognosis of pancreatic cancer.

## 2 METHODS

### 2.1 Clinical specimens

A total of 1850 healthy controls (906 men and 944 women; median age, 45 years; range, 17‐78 years) from the Medical Examination Centre of the Affiliated Hospital of Nantong University between October 2014 and January 2016 were enrolled in the study. They had no history of any basic disease and underwent routine physical examination, including blood, urine and stool tests, chest and abdomen computed tomography (CT) scan and ultrasound of the thyroid, breast and heart. Serum samples were collected to analyse level of galectin‐3 and other indicators.

Serum samples were obtained from 54 male and 48 female patients with pancreatic cancer (35 of them aged <60 years and 67 were ≥60 years) treated at the Affiliated Hospital of Nantong University between September 2014 and June 2016. Pancreatic cancer mentioned in this article was pathologically confirmed adenocarcinoma, whereas neuroendocrine tumours or cystic malignancies of the pancreas were excluded. Patients undergoing surgery were confirmed by post‐operative pathology, whereas those receiving non‐operative treatments were diagnosed by magnetic resonance imaging (MRI) and pathologically confirmed by fine‐needle aspiration. The following entry criteria were applied: (a) patients pathologically proven to have pancreatic adenocarcinoma; (b) obtainable pre‐ and post‐operative blood samples; and (c) informed consent before enrolment. Patients without follow‐up data or with other basic diseases or infection were excluded from the study. Clinical data included patient age, tumour size, clinical stage, lymph node metastasis, distant metastasis and treatment methods. Tumours were detected in the head or neck of the pancreas in 62 patients, whereas the remaining 40 patients had tumours in the body or tail of the pancreas. Tumour diameter was <3 cm in 48 patients and ≥3 cm in 54 patients. Lymph node metastasis and liver metastasis were noted in 40 patients each. Sixty‐eight patients had tumour node metastasis (TNM) stage I/stage II cancer, and 34 patients had TNM stage III/stage IV cancer. Clinical staging of patients was performed according to the International Union Against Cancer TNM classification of malignant tumours.^16^ All samples were divided into two groups as follows: (a) the operative group (36 patients) included 21 patients who underwent radical resection and 15 who underwent palliative resection. Serum samples were collected from all patients to detect galectin‐3 level before and at 1, 3 and 6 months after treatment. One and two patients who underwent radical and palliative resection, respectively, were lost to follow‐up after 3 months. Two patients in each of the two groups were lost within the first half‐year. The follow‐up end‐point was December 2016. (b) The non‐operative group (66 patients) included 17 patients who underwent radiotherapy, 21 who underwent chemotherapy, 10 who underwent interventional therapy and 18 who underwent conservative management. Serum samples were finally collected for galectin‐3 detection before and 1 month after treatment owing to poor medical compliance and loss to flow‐up. The follow‐up end‐point was December 2016.

A total of 200 samples used for prognostic analysis were collected from 118 male and 82 female patients with pancreatic cancer (51 patients were <60 years old and 149 were ≥60 years old) at the Affiliated Hospital of Nantong University from January 2011 to June 2016. The tumours were located in the head or neck of the pancreas in 122 patients and in the body or tail of pancreas in 78 patients. In total, 58 patients showed lymph node metastasis and 84 patients had liver metastasis. In total, 19, 55, 26, and 100 patients had TNM stage I, stage II, stage III and stage IV tumours, respectively. According to treatments, the patients were divided into four groups as follows: (a) 81 patients underwent surgery; (b) 49 underwent radiochemotherapy; (c) 11 underwent interventional therapy (biliary drainage); and (d) 59 underwent conservative management. The follow‐up end‐point was December 2016.

The study was approved by the Medical Ethics Committee of the Affiliated Hospital of Nantong University (2017‐K035) and conducted in accordance with the Declaration of Helsinki. Informed consent was obtained from all patients or their legal guardians prior to the study.

### 2.2 Time‐resolved fluorescence immunoassay (TRFIA)

A 96‐well microplate was coated with an anti‐human galectin‐3 antibody (1:5000 diluted in phosphate‐buffered saline [PBS]; R&D) and incubated at 4°C for 22 hours. Excess of antibody was washed off, and wells were washed thrice with PBS/Tween‐20 (PBST). The microplate was blocked with 10% foetal calf serum at 37°C for 1 hour and washed with PBST. Serum samples and standard samples were added to wells, and the plate was incubated at 37°C for 1 hour. After washing thrice with PBST, each well was treated with 100 μL of a biotinylated polyclonal goat anti‐human galectin‐3 antibody (Detect Antibody; R&D) at 37°C for 1 hour. The well was washed thrice with a wash buffer and then treated with 100 μL of an assay buffer containing Europium‐labelled streptavidin (PE, USA) in the dark at 37°C for 1 hour. After washing four times in wash buffer, each well was treated with 200 μL enhancement solution (PE, USA) and incubated at room temperature (22°C) for 45 minutes.

Samples were analysed using an automatic time‐resolved fluorescence analyzer (PE, USA) at an excitation wavelength of 337 nm and emission wavelength of 615 nm. CurveExpert 1.3 software was used to determine standard curve and calculate the sample concentration.

According to our previous study,^15^ the diagnostic sensitivity for pancreatic cancer was 75.5% and the false‐positive rate was 9.1% when the diagnostic threshold in TRFIA for serum galectin‐3 detection was 3.77 μg/L.

### 2.3 Statistical analysis

Statistical analysis was performed with SPSS 19.0. The chi‐square test or Fisher's exact test was used for any four‐dimensional table test. The measured levels of serum galectin‐3 are presented as median (range). The rank‐sum test was used to analyse the relationship between the groups and clinical parameters. Kaplan‐Meier survival analysis was performed, and survival curves were obtained. Single clinical parameters and survival rates of patients with pancreatic cancer were analysed using the log‐rank test. The effects of different parameters on patient survival were evaluated by a multivariate analysis using the Cox proportional hazard regression model. *P* < .05 was considered statistically significant.

## 3 RESULTS

### 3.1 Serum galectin‐3 levels in healthy controls

The concentrations of serum galectin‐3 in 57 of 1850 healthy controls (range: 3.77‐9.85 μg/L) were higher than those reported in other controls (range: 0.00‐1.03 μg/L; *P* < .05). Analysis of these 57 patients using examination data, other reports and clinical diagnoses showed that 20 of them had thyroid tumours, nine had cardiovascular disease, four had suspected liver cancer, one had suspected pancreatic cancer, and 23 had no other diseases. Among the 57 controls with abnormal galectin‐3 levels, a 51‐year‐old woman with suspected pancreatic cancer showed significantly elevated galectin‐3 level (9.85 μg/L). The results are shown in Table S1.

Clinical test results of this patient with suspected pancreatic cancer were collected. No abnormalities were observed in serum tumour markers or routine blood and urine tests, except that total bilirubin level (21.3 μmol/L) was slightly elevated in the liver function examination. CT and MRI results revealed distal pancreatic duct expansion in the pancreatic neck space, possibly indicating pancreatic cancer (Figure S1A‐B). The patient was admitted to undergo radical resection in the general surgery department of our hospital. Pathological analysis confirmed the diagnosis of moderately differentiated pancreatic carcinoma (Figure S1C).

### 3.2 Relationship between serum galectin‐3 level and clinical parameters in patients with pancreatic cancer before treatment

A total of 102 serum samples collected from patients with pancreatic cancer between September 2014 and June 2016 before treatment were used to analyse the relationship between galectin‐3 level and clinical parameters. Galectin‐3 level showed no significant correlation with clinical parameters such as sex, age, tumour size, tumour location, lymph node metastasis, distant metastasis and TNM stage (*P* > .05; Table 1).

**Table 1:** The correlation of serum galectin‐3 level with clinical parameters in patients with pancreatic cancer before treatment

Parameter	n	Positive (%)	Galectin‐3 (μg/L)	*Z*	*P*
		(>3.77 μg/L)	Median (range)		
Gender					
Male	54	40 (74.1)	4.23 (2.15~23.18)	0.436	.645
Female	48	35 (73.0)	3.86 (1.89~23.04)		
Age					
<60	35	29 (82.8)	3.62 (1.89~20.85)	3.293	.254
≥60	67	52 (77.6)	4.56 (2.12~23.18)		
Tumour size (cm)					
<3	48	36 (75.0)	4.69 (1.93~21.54)	1.895	.472
≥3	54	46 (85.2)	5.53 (2.35~22.67)		
Tumour location					
Head and neck of pancreas	62	40 (64.5)	4.29 (2.03~23.08)	1.241	.582
Body and tail of pancreas	40	30 (75.0)	5.42 (2.15~22.75)		
Liver metastasis					
No	62	52 (83.9)	4.65 (2.53~23.15)	4.294	.173
Yes	40	31 (77.5)	5.26 (2.79~23.18)		
Lymph node metastasis					
No	62	48 (77.4)	3.02 (2.18~21.92)	6.328	.165
Yes	40	29 (72.5)	4.13 (2.26~23.18)		
TNM stage					
I/II	68	52 (76.4)	4.45 (2.19~22.06)	2.915	.326
III/IV	34	28 (82.4)	6.02 (2.52~23.18)		
Treatment					
Supportive treatment	18	12 (80.0)	4.95 (3.79~21.96)	0.253	.782
Radiochemotherapy	38	27 (79.4)	3.87 (2.96~22.13)		
Operation	36	28 (77.8)	3.54 (1.89~20.59)		
Biliary drainage	10	13 (76.5)	4.62 (3.87~23.18)		
CEA level (μg/L)					
<5	52	23 (44.2)	4.12 (2.52~18.35)	1.457	.438
≥5	50	28 (56.0)	4.46 (3.16~22.35)		
CA19‐9 level (U/mL)					
<37	42	20 (47.4)	4.02 (1.89~21.37)	3.268	.251
≥37	60	40 (66.7)	5.12 (2.45~23.18)		

### 3.3 Serum galectin‐3 levels before and after radical resection and palliative resection

**Figure 1:**
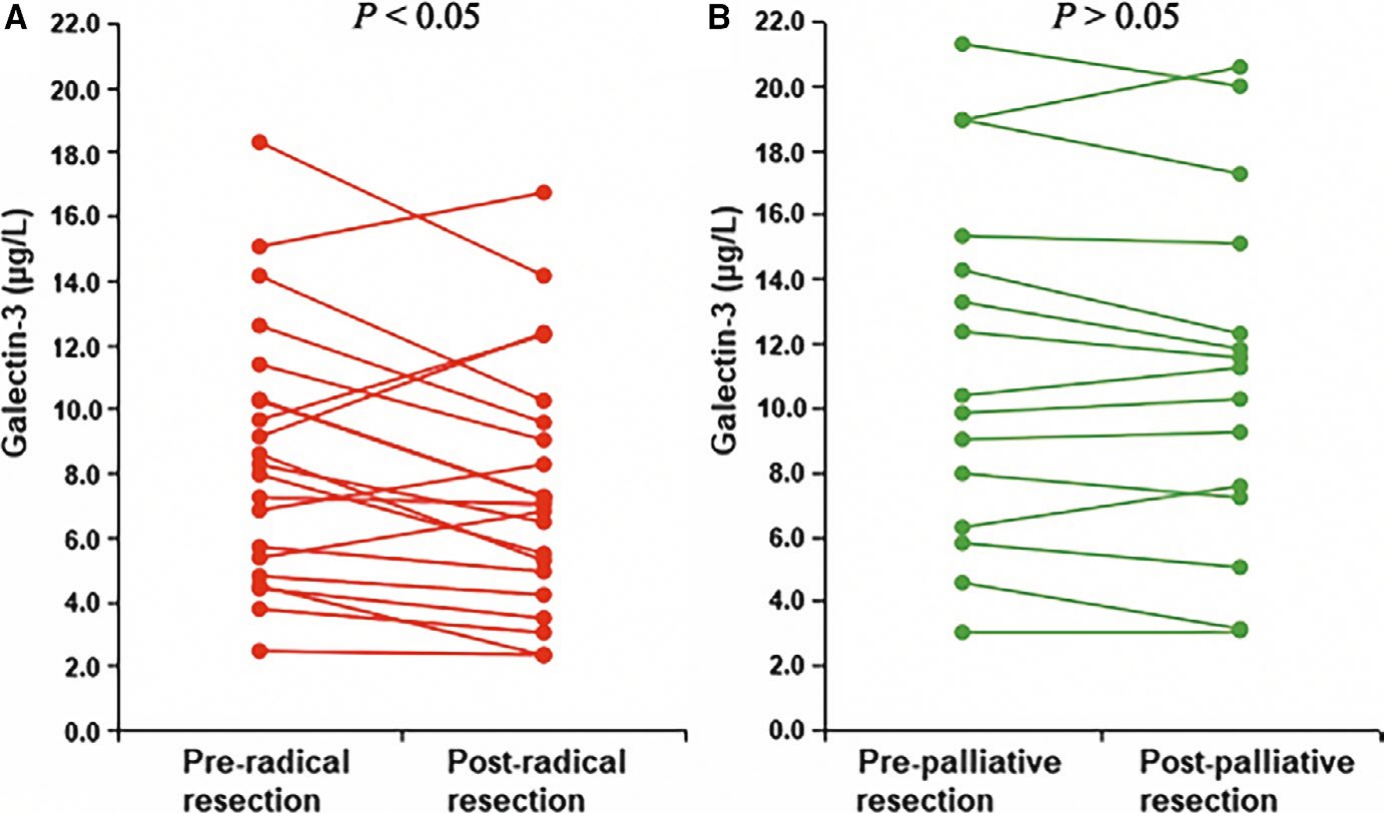
The trend of monthly concentration changes after A, radical resection (*P* < .05) and B, palliative resection (*P* > .05)

A total of 36 patients underwent surgical resection (radical resection, n = 21, palliative resection, n = 15). Galectin‐3 levels before treatment and 1, 3 and 6 months after resection were detected to evaluate any relationship with the curative effect. No observable difference was noted between the two groups before treatment (Table S2, *P* > .05). Galectin‐3 levels dramatically decreased 1 month after radical resection, and the difference was still statistically significant at 3 and 6 months post‐operatively compared with pre‐operative levels (Table S2, Figure 1A, *P* < .05). However, there was no remarkable difference between the three post‐operative time‐points (Table S2, *P* < .05). No significant change in galectin‐3 levels was observed before and after palliative resection (Table S2, Figure 1B, *P* > .05).

### 3.4 Relationship between galectin‐3 level and metastasis or recurrence after radical resection

Among 21 patients who underwent radical resection, five showed no decrease in galectin‐3 levels. Galectin‐3 expression decreased 1 month after resection in 16 patients. All 21 patients were followed up for half a year. Metastasis or recurrence was observed in four patients with decreased galectin‐3 levels and four without. Metastasis or recurrence rate was higher among patients without decrease in galectin‐3 levels than in those with decreased galectin‐3 levels (*P* < .05, Table 2).

**Table 2:** The relationship between serum galectin‐3 levels and tumour metastasis or recurrence 1 mo after radical resection

Galectin‐3 level	Case, n	Metastasis or recurrence (%)
No decrease	5	4 (80.0)*
Decrease	16	4 (25.0)

* *P* < .05.

### 3.5 Survival analysis of patients undergoing surgical resection

**Figure 2:**
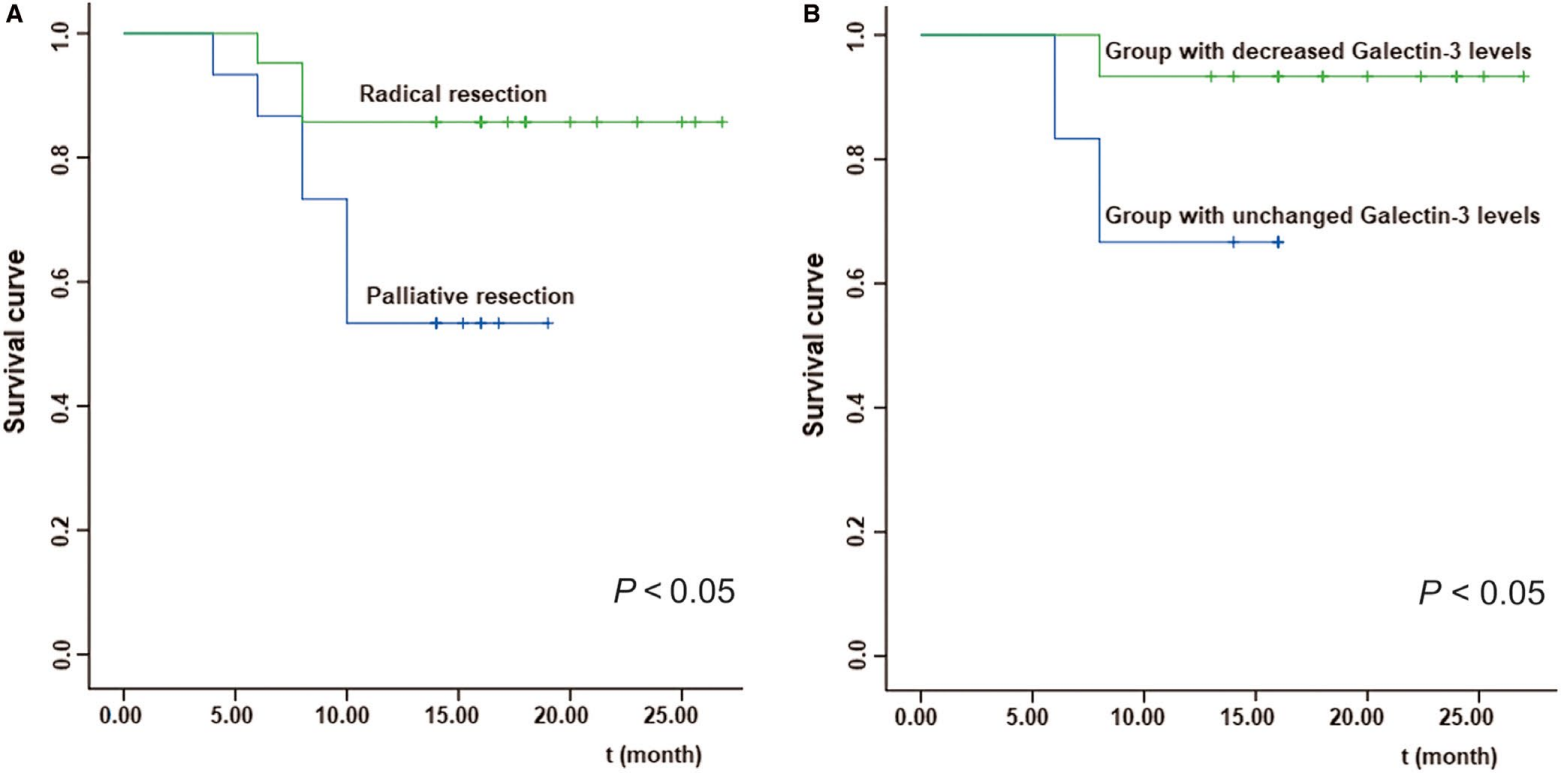
Survival curve. A, Comparison of survival rates after radical resection and palliative resection (*P* < .05). B, Comparison of survival rates between patients with decreased serum galectin‐3 level and with no change in galectin‐3 level (*P* < .05)

Considering the 36 patients from the operative group, survival time was longer in patients who underwent radical resection than in those who underwent palliative resection (Kaplan‐Meier analysis; Figure 2A, *P* < .05). Among the 21 patients who underwent radical resection, patients with reduced galectin‐3 levels survived longer than those with increased or unchanged galectin‐3 levels (Figure 2B, *P* < .05).

### 3.6 Comparison of serum galectin‐3 levels before and after treatment in the non‐operative group

Treatment was considered effective in 31 patients and ineffective in 35 of 66 patients evaluated 1 month after treatment (Table S3). No significant difference in galectin‐3 levels was detected between the two groups of patients either before or after treatment. Further, galectin‐3 levels did not significantly change after non‐operative treatment (*P* > .05, Table S3, Figure S2).

### 3.7 Correlation between median survival time and clinical parameters of patients with pancreatic cancer

For the 200 patients with pancreatic cancer who were followed up, the median survival time was 5.5 months. Survival time correlated with clinical parameters (pancreatic cancer patient age, serum galectin‐3 level, CA19‐9 and CEA levels, TNM stage, liver or lymph node metastasis, and treatment; *P* < .05, Table S4).

**Figure 3:**
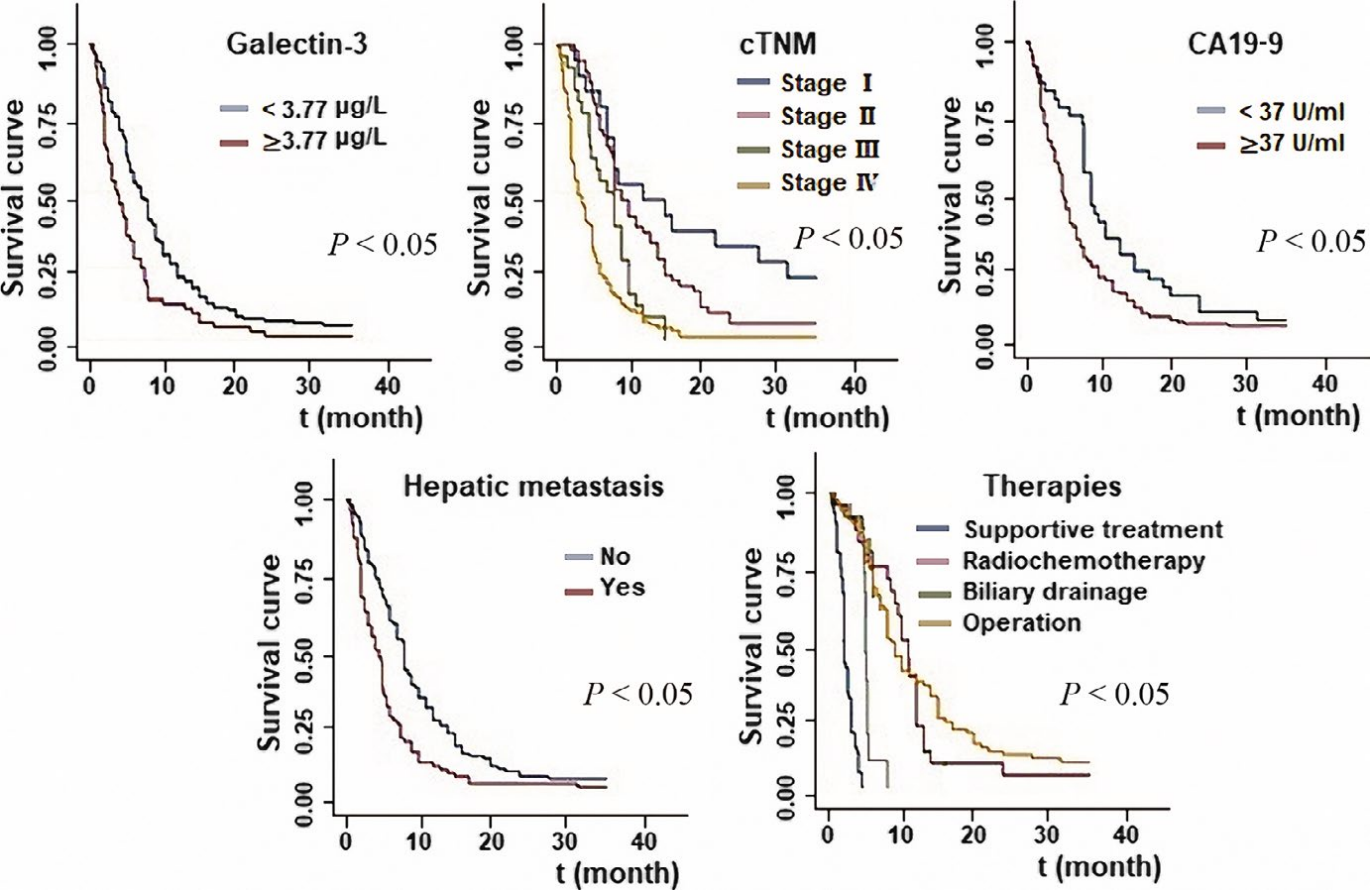
Survival curve. The correlation between significant clinical parameters (serum galectin‐3 level, TNM stage, CA19‐9 levels, hepatic metastasis and treatment) and survival rate was determined. *P* < .05

Single‐factor and multivariate Cox analyses showed that serum galectin‐3 and CA19‐9 levels, TNM stage, liver metastasis and treatment were independent prognostic factors for survival in patients with pancreatic cancer. At the time of diagnosis, the risk of death increased in patients with abnormal serum galectin‐3 and CA19‐9 levels, and the hazard ratio (HR) of galectin‐3 (HR = 1.97; 95% confidence interval [CI]: 0.89, 3.58; *P* = .005) was higher than that of CA19‐9 (HR = 1.93; 95% CI: 1.85, 1.98; *P* = .006; Table 3). The correlation between galectin‐3 level and survival rate was determined in 200 patients by Kaplan‐Meier analysis. The higher the serum galectin‐3 level, the shorter was the survival time (Figure 3).

**Table 3:** Univariate and multivariate Cox analysis of the overall survival

Parameter	Univariate analysis		Multivariate analysis	
	HR (95% CI)	*P*	HR (95% CI)	*P*
Gender	1.08 (0.81, 1.44)	.587		
Age	1.41 (1.02, 1.95)	.039	1.31 (0.92, 1.87)	.131
Diabetes	1.03 (0.73, 1.45)	.862		
Tumour location	1.34 (1.05, 1.79)	.048		
Liver metastasis	1.81 (1.35, 2.42)	<.001	1.39 (1.02, 1.94)	.019
Lymph node metastasis	1.78 (1.31, 2.43)	<.001	1.25 (0.88, 1.78)	.207
TNM stage				
I	1.00		1.00	
II	1.60 (0.91, 2.87)	.111	1.85 (1.02, 3.35)	.043
III	2.86 (1.48, 5.53)	.002	2.74 (1.36, 5.53)	.005
IV	4.58 (2.60, 8.07)	<.001	3.29 (1.65, 6.55)	.001
Treatments				
Supportive treatment	1.00		1.00	
Radiochemotherapy	0.04 (0.02, 0.08)	<.001	0.06 (0.03, 0.13)	<.001
Operation	0.03 (0.02, 0.06)	<.001	0.05 (0.02, 0.11)	<.001
Biliary drainage	0.16 (0.08, 0.35)	<.001	0.14 (0.06, 0.32)	<.001
CEA level	1.82 (1.37, 2.43)	<.001	1.23 (0.54, 2.71)	.143
CA19‐9 level	1.64 (1.13, 2.39)	.009	1.93 (1.85, 1.98)	.006
Galectin‐3 level	1.66 (0.82, 2.96)	.008	1.97 (0.89, 3.58)	.005

**Figure 4:**
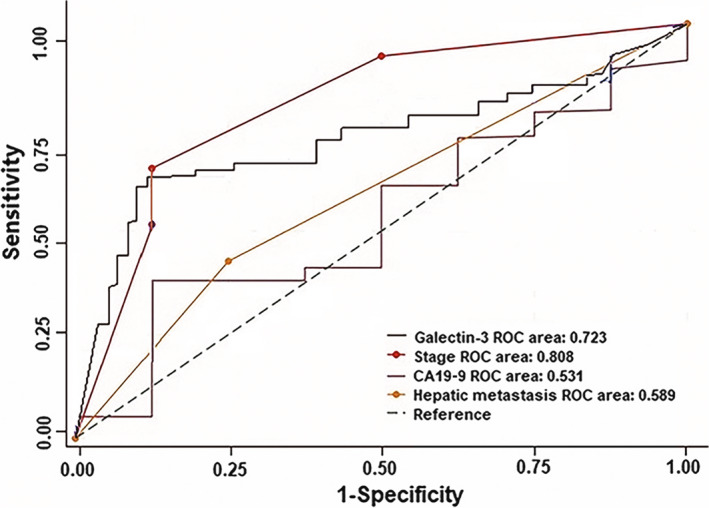
ROC curve of the independent prognostic factors of three‐year survival in pancreatic cancer patients

A receiver operating characteristic (ROC) curve was constructed using SPSS 19.0. We used 3.77 μg/L as the threshold and found that the sensitivity, specificity and Youden index of galectin‐3 to predict survival outcome in patients with pancreatic cancer were 74.8%, 90.2% and 65%, respectively. The sensitivity, specificity and Youden index were 89.7%, 86.1% and 75.8%, respectively, using TNM III. These results suggest that galectin‐3 level and TNM classification (cTNM) had high predictive values for survival outcome in patients with pancreatic cancer (Table S5; Figure 4).

## 4 DISCUSSION

Poor prognosis and high mortality rate in patients with pancreatic cancer are largely associated with the lack of early diagnosis. The 5‐year survival rate of pancreatic cancer is <5%.^17^ Therefore, the identification of potential biomarkers for early diagnosis and monitoring of post‐operative results is the focus of pancreatic cancer research.

At present, CA19‐9 is a common clinical tumour marker for pancreatic cancer. Kim *et al* ^18^ reported that the level of CA19‐9 was >37 units/mL in 1036 of 70 490 patients, but only four were diagnosed with pancreatic cancer. The positive predictive value of CA19‐9 was only 0.9% in asymptomatic populations; therefore, CA19‐9 may not be suitable for screening patients with pancreatic cancer. Ballehaninna *et al* ^19^ indicated the applicability of CA19‐9 to evaluate surgical resectability of pancreatic cancer at a concentration cut‐off of 100 units/mL. The positive predictive value of tumour resectability was approximately 60%‐80% in patients with CA19‐9 levels lower than this cut‐off value. However, CA19‐9 is still not an ideal diagnostic and evaluation indicator for pancreatic cancer. Therefore, multiple studies have been conducted to identify novel biomarkers that provide earlier prediction or more accurate prognosis for pancreatic cancer.^20^, ^21^ Increased miR‐223 level is thought to contribute to the poor prognosis of patients with pancreatic cancer.^22^ The expression of interleukin‐6 could also be used to predict the survival time of patients with pancreatic cancer with liver metastases.^23^

Galectin‐3 was overexpressed in pancreatic cancer tissues in our previous study.^14^, ^15^ Song et al also performed immunohistochemical staining of human pancreatic tissue microarrays with anti–galectin‐3 antibody and reported the up‐regulated expression of galectin‐3 in human pancreatic tumour tissues.^24^ In the present study, the serum concentration of galectin‐3 in one healthy control (9.85 μg/L) was significantly higher than that of other controls. This control had no abnormal level of CA19‐9 or CEA and no relative symptoms of discomfort. Considering that pancreatic tumour was noted in imaging reports, moderately differentiated adenocarcinoma of the pancreas was further confirmed by post‐operative pathology. The above data suggest that galectin‐3 may provide an effective reference value for the screening of pancreatic cancer, especially in high‐risk groups. It worth noting that among 57 of 1850 healthy controls with high level of galectin‐3, many other diseases other than pancreatic cancer are galectin‐3 high including suspected liver cancer, cardiovascular disease, thyroid disease and even no other diseases. But as we know, other tumour markers such as AFP, CEA and CA19‐9 can also slightly elevate in inflammatory diseases such as hepatitis and pancreatitis, but their properties as independent tumour markers are not affected.

In our previous research, ROC analysis showed that the diagnostic power of galectin‐3 for pancreatic cancer greatly improved in combination with the conventional tumour markers CA19‐9 and CEA.^15^ Consistent with our previous findings, our current study indicated no significant correlation between the serum level of galectin‐3 and clinicopathological parameters (*P* > .05). In particular, galectin‐3 expression showed no significant correlation with lymph node metastasis, distant metastasis or TNM stage, indicating that galectin‐3 is an important serum tumour marker for the early diagnosis of pancreatic cancer.

In the present study, serum galectin‐3 level was dynamically monitored in 36 patients, and no significant difference was observed between the two groups before treatment (*P* > .05). Compared to the pre‐operative levels, serum galectin‐3 levels in patients who underwent radical resection decreased 1 month after surgery (*P* < .05). No significant difference was observed between pre‐operative and post‐operative serum galectin‐3 levels in patients who underwent palliative resection (*P* > .05). The survival time of patients who underwent radical resection was longer than that of patients who underwent palliative resection. The lack of any decrease in galectin‐3 level may contribute to these results. Some researchers assume that mucin 4 (MUC4), a high‐molecular‐weight glycoprotein aberrantly expressed by pancreatic carcinoma cells, helps in the docking of tumour cells on the endothelial surface. During cancer progression, galectin‐3‐MUC4 interaction–mediated clustering of MUC4 may expose the surface adhesion molecules, which in turn promotes a stronger attachment (locking) of tumour cells to the endothelial surface.^25^ Further, galectin‐3 is known to interact with and mediate the expression of many proteins, contributing to epithelial‐to‐mesenchymal transition (EMT) during tumour cell migration and invasion.^26^ Based on these findings, we analysed the relationship between galectin‐3 level and metastasis or recurrence after radical resection and performed survival analysis for patients undergoing surgical resection. Our findings revealed that patients without decreased galectin‐3 levels after radical resection had higher rate of recurrence and metastasis and worse prognosis (*P* < .05). In patients who received non‐operative treatment (including radiotherapy, chemotherapy, interventional therapy and conservative management), there was no prominent difference in serum galectin‐3 levels before and 1 month after treatment (*P* > .05), indicating the unsuitability of galectin‐3 for efficacy evaluation in these patients. The lack of any significant decrease in galectin‐3 level may be related to the presence of tumour, despite palliative resection or chemoradiotherapy. Another reason may be the chemoresistance of cancer stem cells.^26^ Persistent tumour combined with galectin‐3–induced chemoresistance constitutes a vicious circle.

To determine whether the serum level of galectin‐3 exhibits prognostic relevance, we assessed the effect of serum galectin‐3 and CA19‐9 levels, TNM stage, liver metastasis and treatment methods. All of these parameters served as independent prognostic factors of survival time in 200 patients with pancreatic cancer. Once diagnosed with pancreatic cancer, the risk of death in patients with abnormal serum galectin‐3 and CA19‐9 levels was high (risk of galectin‐3 abnormality was higher than that with CA19‐9 abnormality). Furthermore, the higher the serum galectin‐3 level, the shorter was the survival time. The sensitivity, specificity and Youden index of serum galectin‐3 level to predict survival outcome in patients with pancreatic cancer were 74.8%, 90.2% and 65%, respectively, and these values were 89.7%, 86.1% and 75.8%, respectively, for TNM stage III. Thus, galectin‐3 and cTNM have high predictive values for survival outcome in patients with pancreatic cancer.

This study analysed the relationship between serum galectin‐3 level and clinicopathological parameters, changes in galectin‐3 concentration before and after treatment, and effects of galectin‐3 on the prognosis of patients with pancreatic cancer. Our results suggest that galectin‐3 is likely to become a biomarker for the screening and early diagnosis of pancreatic cancer and may serve as an independent prognostic indicator for patients with pancreatic cancer. However, the sample size of patients with pancreatic cancer in our study was not large, especially for each subgroup. Even fewer patients had undergone radical resection. Our results gave a great indication. Further studies with added number of cases are therefore warranted to confirm these results.

## CONFLICT OF INTEREST

The authors have declared that no competing interest exists.

## AUTHOR CONTRIBUTIONS

Nan Yi: Study design, conduction of experiments, analysis and interpretation of the data, sample and clinical data collection, and writing of the manuscript. Xuying Zhao: Study design, conduction of experiments, analysis and interpretation of the data, sample and clinical data collection, and writing of the manuscript. Jianhua Chen: Study design, writing of the manuscript, and analysis and interpretation of the data. Mingbing Xiao: Study design, analysis and interpretation of the data, and writing of the manuscript. Minxue Xu: Conduction of experiments, and analysis and interpretation of the data. Yujie Jiao: Conduction of experiments. Tianyang Qian: Conduction of experiments, analysis and interpretation of the data, and sample and clinical data collection. Jie Ji: Analysis and interpretation of the data. Feng Jiang: Sample and clinical data collection. Shengze Zhu: Sample and clinical data collection. All authors: Revision and approval of the final manuscript.

## Supporting information

Figure S1

Figure S2

Table S1

Table S2

Table S3

Table S4

Table S5

## Data Availability

All data generated or analysed during this study are included in this article.
