# Supplementary material for: Serum galectin‐3 as a biomarker for screening, early diagnosis, prognosis and therapeutic effect evaluation of pancreatic cancer
Source: J Cell Mol Med. 2020 Sep 4;24(19):11583–91. doi: 10.1111/jcmm.15775 (PMC7576229; doi:10.1111/jcmm.15775)
Supplement: Supplementary file 1 — Figure S1 [file JCMM-24-11583-s001.docx]

**Supplementary Figure 1**


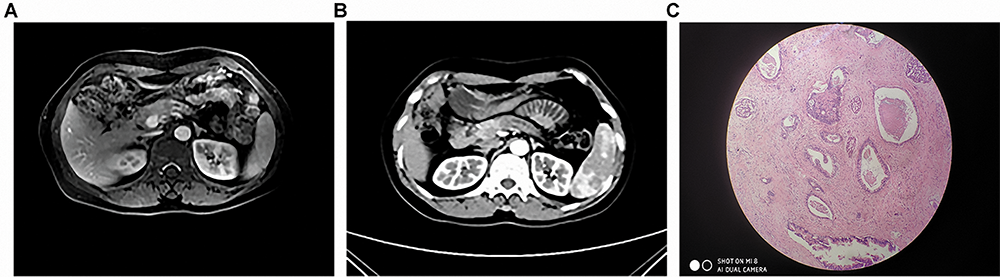


Supplementary Figure 1. (A) A contrast-enhanced computed tomography (CT) scan of the upper abdomen, (B) magnetic resonance cholangiopancreatography (MRCP) demonstrated that pancreatic neck space was accompanied by distal pancreatic duct expansion, (C) pathological diagnosis confirmed that the tumour was a moderately differentiated pancreatic adenocarcinoma.
