# Supplementary material for: Serum galectin‐3 as a biomarker for screening, early diagnosis, prognosis and therapeutic effect evaluation of pancreatic cancer
Source: J Cell Mol Med. 2020 Sep 4;24(19):11583–91. doi: 10.1111/jcmm.15775 (PMC7576229; doi:10.1111/jcmm.15775)
Supplement: Supplementary file 2 — Figure S2 [file JCMM-24-11583-s002.docx]

**Supplementary Figure 2**


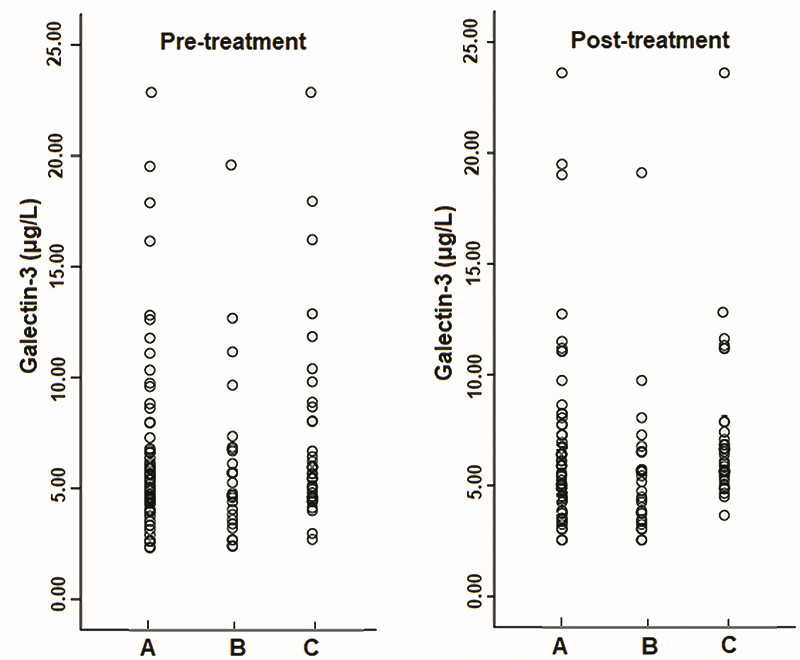


Supplementary Figure 2. Scatter plots of serum galectin-3 levels in patients before and after non-operative treatment. (A) No surgery. (B) Effective treatment. (C) Ineffective treatment.
