# Supplementary material for: Serum galectin‐3 as a biomarker for screening, early diagnosis, prognosis and therapeutic effect evaluation of pancreatic cancer
Source: J Cell Mol Med. 2020 Sep 4;24(19):11583–91. doi: 10.1111/jcmm.15775 (PMC7576229; doi:10.1111/jcmm.15775)
Supplement: Supplementary file 3 — Table S1 [file JCMM-24-11583-s003.docx]

**Supplementary Table 1. The concertration of galectin-3 (μg/L)**

| Types | n | galectin-3 |
| --- | --- | --- |
| Suspected pancreatic cancer | 1 | 9.85 |
| Suspected liver cancer | 4 | 4.43 (3.91～5.78)^*^ |
| Cardiovascular disease | 9 | 4.26 (3.79～5.22) ^*^ |
| Thyroid disease | 20 | 4.08 (3.83～4.47) ^*^ |
| No other diseases | 23 | 3.83 (3.77～3.92) ^*^ |
| Others | 1793 | 0.65 (0.00～1.03) |

**P*<0.05 vs. others
