# Supplementary material for: Serum galectin‐3 as a biomarker for screening, early diagnosis, prognosis and therapeutic effect evaluation of pancreatic cancer
Source: J Cell Mol Med. 2020 Sep 4;24(19):11583–91. doi: 10.1111/jcmm.15775 (PMC7576229; doi:10.1111/jcmm.15775)
Supplement: Supplementary file 4 — Table S2 [file JCMM-24-11583-s004.docx]

**Supplementary Table 2. Galectin-3 levels in the patients before and after surgery**

| **Treatment** | **Radical resection** | | **Palliative resection** | |
| --- | --- | --- | --- | --- |
|  | **n** | **Galectin-3 (μg/L)** | **n** | **Galectin-3 (μg/L)** |
| Preoperative | 21 | 5.36 (1.89～18.29) ^a^ | 15 | 5.42 (3.02～20.59) ^c^ |
| One month postsurgery | 21 | 4.32 (1.80～16.72) ^b^ | 15 | 5.39 (3.13～20.56) ^c^ |
| Three months postsurgery | 20 | 4.27 (2.08～17.73 )^b^ | 13 | 5.43 (3.26～22.45) ^c^ |
| Six months postsurgery | 18 | 4.79 (2.40～18.65) ^b^ | 11 | 5.64 (4.07～21.73) ^c^ |

a *vs.* b, *P*<0.05; pairs among b, *P*>0.05; a *vs.*c, *P*>0.05; pairs among c, *P*>0.05
