# Supplementary material for: Serum galectin‐3 as a biomarker for screening, early diagnosis, prognosis and therapeutic effect evaluation of pancreatic cancer
Source: J Cell Mol Med. 2020 Sep 4;24(19):11583–91. doi: 10.1111/jcmm.15775 (PMC7576229; doi:10.1111/jcmm.15775)
Supplement: Supplementary file 5 — Table S3 [file JCMM-24-11583-s005.docx]

| **Supplementary Table 3. Serum galectin-3 level in the patients before and after non operative treatment** | | | | |
| --- | --- | --- | --- | --- |
| **Treatment** | **effective** | | **ineffective** | |
|  | n | Galectin-3 (μg/L) | n | Galectin-3 (μg/L) |
| Before treatment | 31 | 6.52 (2.96～20.73)^a^ | 35 | 6.73 (3.06～23.18)^c^ |
| After treatment | 28 | 6.27 (3.05～19.62)^b^ | 32 | 7.08 (3.55～23.23)^d^ |
| All *P*>0.05, a *vs.* b; c *vs.* d; a *vs.* c; b *vs.* d. | | | | |
