# Supplementary material for: Serum galectin‐3 as a biomarker for screening, early diagnosis, prognosis and therapeutic effect evaluation of pancreatic cancer
Source: J Cell Mol Med. 2020 Sep 4;24(19):11583–91. doi: 10.1111/jcmm.15775 (PMC7576229; doi:10.1111/jcmm.15775)
Supplement: Supplementary file 6 — Table S4 [file JCMM-24-11583-s006.docx]

**Supplementar Table 4. Correlation between median survival time and clinical parameters of patients with pancreatic cancer**

| **Parameter`** | **n** | **%** | **Median survival time (95%CI)** | |
| --- | --- | --- | --- | --- |
|  |  |  | (Month) | ***P*** |
| **Gender** |  |  |  |  |
| Male | 118 | 59.0% | 5.50 (4.87,6.12) | 0.573 |
| Female | 82 | 41.0% | 5.00 (3.58,6.41) |  |
| **Age** |  |  |  |  |
| <60 | 51 | 25.5% | 8.00 (5.81,10.18) | 0.031 |
| ≥60 | 149 | 74.5% | 5.00 (4.40,5.59) |  |
| **Diabetes** |  |  |  |  |
| No | 158 | 79.0% | 5.50 (4.75,6.25) | 0.857 |
| Yes | 42 | 21.0% | 5.50 (3.65,7.35) |  |
| **Tumor location** |  |  |  |  |
| Head and neck of pancreas | 122 | 61.0% | 7.00 (5.34,8.65) | 0.069 |
| Body and tail of pancreas | 78 | 39.0% | 5.00 (2.91,6.98) |  |
| **Liver metastasis** |  |  |  |  |
| No | 116 | 58.0% | 8.00 (7.08,8.92) | <0.001 |
| Yes | 84 | 42.0% | 4.00 (3.10,4.89) |  |
| **Lymph node metastasis** |  |  |  |  |
| No | 142 | 71.0% | 6.50 (5.27,7.72) | <0.001 |
| Yes | 58 | 29.0% | 3.00 (1.51,4.49) |  |
| **TNM stage** |  |  |  |  |
| Ⅰ | 19 | 9.5% | 12.00 (2.04,21.95) | <0.001 |
| Ⅱ | 55 | 27.5% | 9.00 (7.55,10.45) |  |
| Ⅲ | 26 | 13.0% | 6.00 (3.02,8.99) |  |
| Ⅳ | 100 | 50.0% | 3.00 (1.95,4.05) |  |
| **Treatment** |  |  |  |  |
| Supportive treatment | 59 | 29.5% | 2.00 (1.89,2.11) | <0.001 |
| Radiochemotherapy | 49 | 24.5% | 6.80 (5.26,9.44) |  |
| Operation | 81 | 40.5% | 8.50 (7.48,10.81) |  |
| Biliary drainage | 11 | 5.5% | 5.20 (4.98,5.51) |  |
| **CEA level** |  |  |  |  |
| <5 μg/L | 108 | 54.0% | 7.50 (6.69,8.30) | <0.001 |
| ≥5 μg/L | 92 | 46.0% | 4.00 (2.49,5.50) |  |
| **CA19-9 level** |  |  |  |  |
| <37 U/ml | 36 | 18.0% | 9.00 (7.53,10.46) | 0.006 |
| ≥37 U/ml | 164 | 82.0% | 5.00 (4.53,5.46) |  |
| **Serum galectin-3 level** |  |  |  |  |
| <3.77 μg/L | 48 | 24.0% | 10.5 (5.02,15.02) | <0.001 |
| ≥3.77 μg/L | 152 | 76.0% | 4.13 (2.12,5.31) |  |
