# Supplementary material for: Serum galectin‐3 as a biomarker for screening, early diagnosis, prognosis and therapeutic effect evaluation of pancreatic cancer
Source: J Cell Mol Med. 2020 Sep 4;24(19):11583–91. doi: 10.1111/jcmm.15775 (PMC7576229; doi:10.1111/jcmm.15775)
Supplement: Supplementary file 7 — Table S5 [file JCMM-24-11583-s007.docx]

**Supplementary Table 5. Prognostic value of prognostic factors in 3 year survival outcomes**

| **Parameter** | **AUC** | ***P*** | **Cutoff value** | **Sensitivity (%)** | **Specificity (%)** | **Yuden index (%)** |
| --- | --- | --- | --- | --- | --- | --- |
| **TNM** | 0.808（0.640,0.980） | <0.001 | Ⅲ | 89.7 | 86.1 | 75.8 |
| **Galectin-3** | 0.723（0.673,0.773） | <0.001 | 3.77 | 74.8 | 90.2 | 65.0 |
| **Liver metastasis** | 0.588（0.470,0.630） | 0.089 | 1.00 (Yes) | 68.8 | 71.4 | 40.2 |
| **CA19-9 level** | 0.531（0.330,0.730） | 0.114 | 38.31 | 56.4 | 62.5 | 18.9 |
